# Network Topology Can Explain Differences in Pleiotropy Between *Cis-* and *Trans*-regulatory Mutations

**DOI:** 10.1093/molbev/msac266

**Published:** 2022-12-12

**Authors:** Pétra Vande Zande, Patricia J Wittkopp

**Affiliations:** Department of Molecular, Cellular, and Developmental Biology, University of Michigan, Ann Arbor, Michigan, USA; Department of Molecular, Cellular, and Developmental Biology, University of Michigan, Ann Arbor, Michigan, USA; Department of Ecology and Evolutionary Biology, University of Michigan, Ann Arbor, Michigan, USA

**Keywords:** gene expression, regulatory network, pleiotropy, *Saccharomyces cerevisiae*

## Abstract

A mutation's degree of pleiotropy (i.e., the number of traits it alters) is predicted to impact the probability of the mutation being detrimental to fitness. For mutations that impact gene expression, mutations acting in *cis* have been hypothesized to generally be less pleiotropic than mutations affecting the same gene's expression in *trans*, suggesting that *cis*-regulatory mutations should be less deleterious and more likely to fix over evolutionary time. Here, we use expression and fitness data from *Saccharomyces cerevisiae* gene deletion strains to test these hypotheses. By treating deletion of each gene as a *cis*-regulatory mutation affecting its own expression and deletions of other genes affecting expression of this focal gene as *trans*-regulatory mutations, we find that *cis*-acting mutations do indeed tend to be less pleiotropic than *trans*-acting mutations affecting expression of the same gene. This pattern was observed for the vast majority of genes in the data set and could be explained by the topology of the regulatory network controlling gene expression. Comparing the fitness of *cis*- and *trans*-acting mutations affecting expression of the same gene also confirmed that *trans*-acting deletions tend to be more deleterious. These findings provide strong support for pleiotropy playing a role in the preferential fixation of *cis*-regulatory alleles over evolutionary time.

## Introduction

According to evolutionary theory, the extent of a mutation's pleiotropic effects, or the number of traits it impacts, is an important factor in the net fitness effect of that mutation (Kimura and Ohta 1974; Wagner and Zhang 2011; Paaby and Rockman 2013a). Specifically, as the number of traits that a mutation influences increases, the probability of that mutation having a positive fitness effect decreases. Therefore, mutations that influence many traits simultaneously (i.e., are more pleiotropic) are predicted to be less likely to have a net positive fitness effect than mutations that affect fewer traits (i.e., are less pleiotropic) (Fisher 1930; Pavlicev and Wagner 2012). The theoretical relationship between pleiotropy and fitness is difficult to test empirically because distinguishing between distinct traits can be difficult and because pleiotropy can be defined differently depending on the context in which it is being assayed (Paaby and Rockman 2013a, 2013b; Zhang and Wagner 2013). Within the context of the evolution of gene expression, a mutation's pleiotropic effects can be estimated as the number of genes whose expression is influenced by that mutation. Using this definition of pleiotropy, a few studies have identified a negative relationship between the pleiotropic effects of a mutation and the fitness effects of that mutation for a small number of genes in the baker's yeast *Saccharomyces cerevisiae* (Featherstone and Broadie 2002; Vande Zande et al. 2022). This relationship between a mutation's pleiotropic effects on gene expression and the mutation's fitness effect could potentially be important in determining which regulatory mutations are most likely to contribute to expression divergence. However, whether there are systematic differences between the pleiotropic effects of different classes of regulatory mutations is an open question.

Regulatory mutations can be classified as either *cis*- or *trans*-acting relative to a specific gene whose expression they influence, called the focal gene. *Cis*-regulatory mutations cause an allele-specific change in the focal gene's expression and tend to be located close to the focal gene. By contrast, *trans*-regulatory mutations affect gene expression via a diffusible factor (e.g., protein, RNA), such that they can affect both alleles of the focal gene in a diploid cell. At a genomic level, studies using allele-specific expression to estimate the relative contribution of *cis*- and *trans*-acting changes to regulatory evolution in fruit flies (*Drosophila*) have reported a greater contribution of *cis*- than *trans*-acting variation to expression differences between than within species (Wittkopp et al. 2008; McManus et al. 2010; Coolon et al. 2014), suggesting that *cis*-regulatory divergence might accumulate preferentially over evolutionary time. This pattern was seen even more strongly when comparing strains and species of *Saccharomyces* yeast (Metzger et al. 2017), suggesting the preferential accumulation of *cis*-regulatory divergence might be a common feature of eukaryotic regulatory evolution. Such a pattern could be driven by natural selection favoring the fixation of *cis*-regulatory mutations or disfavoring fixation of *trans*-regulatory mutations (Emerson et al. 2010; Schaefke et al. 2013; Coolon et al. 2015). One commonly hypothesized explanation for this preferential fixation is that *cis*-regulatory mutations are less pleiotropic than *trans*-regulatory mutations, and are therefore less likely to be removed by negative selection.

Why would *cis*-regulatory mutations be less pleiotropic than *trans*-regulatory mutations affecting expression of the same gene? One possible explanation is that a *trans*-regulatory mutation influences the expression of the focal gene, which in turn influences the expression of other genes similarly to a *cis*-regulatory mutation, while also having additional effects on other genes in parallel. If this is generally true of *trans*-regulatory mutations, they would necessarily influence the expression of more genes, and be more pleiotropic, than a *cis*-regulatory mutation that affects expression of the same focal gene. We recently tested this model for the focal gene *TDH3* in *S. cerevisiae* and found that indeed *trans*-regulatory mutations are more pleiotropic than *cis*-regulatory mutations of similar effect size, although the effects of *trans*-regulatory mutations on downstream genes were more complex than the simple model above would suggest (Vande Zande et al. 2022). In addition, this simple model would only hold for a relatively simple, branching regulatory network with few interconnections and loops. In contrast, the regulatory network of *S. cerevisiae* is highly interconnected and contains an abundance of feedback and feedforward loops (Kemmeren et al. 2014). The complexity of the regulatory network suggests that *trans*-regulatory mutations might not always be more pleiotropic than *cis*-regulatory mutations affecting expression of the same focal gene.

These observations left us with two questions that we address here: does the pattern of greater pleiotropy for *trans*-regulatory mutations than *cis*-regulatory mutations affecting expression of the same focal gene reported previously for *TDH3* hold for other genes in the yeast genome? And if so, why? To address these questions, we took advantage of a large set of gene expression profiles for gene deletions in *S. cerevisiae* (Kemmeren et al. 2014). Considering each deleted gene to be a focal gene, the deletion of the gene itself acts as a *cis*-regulatory mutation for that gene. Deletions of other genes that result in a change in expression of the focal gene are *trans*-regulatory mutations. By treating each deleted gene in turn as the focal gene, we investigated the pleiotropic effects of deletions occurring in *cis* and *trans* for a total of 748 focal genes. We find that for the vast majority of these focal genes, *trans*-regulatory mutations tend to be more pleiotropic than *cis*-regulatory mutations. We find that this difference in pleiotropy is not explained by the effects of *cis*-regulatory mutations simply being a subset of the effects of *trans*-regulatory mutations. Rather, we show that the overall degree distribution of the regulatory network can explain this pattern of greater pleiotropy for *trans*-regulatory mutations relative to a *cis*-regulatory mutation affecting expression of the same focal gene. Furthermore, because greater pleiotropy is correlated with a greater fitness cost, *cis*-regulatory mutations are indeed less costly than *trans*-regulatory mutations for the majority of focal genes analyzed. These findings provide strong support for the hypothesis that *cis*-regulatory changes accumulate preferentially over evolutionary time because *trans*-regulatory mutations tend to have higher pleiotropy and fitness costs than *cis*-regulatory mutations affecting expression of the same focal gene.

## Results

### Identification of *Cis*- and *Trans*-regulatory Mutations for 754 Focal Genes

To quantify the pleiotropic effects on gene expression of *cis*- and *trans*-regulatory mutations for many focal genes across the genome, we used a large compendium of gene expression profiles, consisting of microarray data measuring expression levels of 6,123 genes in 1,484 single gene deletion mutations in the baker's yeast *S. cerevisiae* (Kemmeren et al. 2014). We considered each deleted gene in turn to be the focal gene. Using the significance and fold-change thresholds used in the original publication of these data (*P*-value < 0.05, fold-change > 1.7, see Materials and Methods), we excluded focal genes (gene deletion strains) that did not show a significant decrease in expression of the deleted gene to ensure we only considered focal genes which were correctly deleted and were expressed in the wild-type strain under assay conditions. This removed 129 focal genes, resulting in measurements of 6,123 genes for a total of 1,355 focal genes. In addition, we can only compare *cis*- and *trans*-regulatory mutations for focal genes for which we can identify at least one *trans*-regulatory deletion. We therefore also removed from our set of gene deletion strains any gene whose expression was not assayed by the microarray (82 deletions) or whose expression was not significantly changed in at least one other deletion strain (525 deletions). This resulted in a final number of 748 focal genes. This set of focal genes contains genes classified in all functional categories present in the original data set, including cell cycle regulators, chromatin factors, gene-specific transcription factors, protein kinases, and genes of unknown function, among others (supplementary Table S1, Supplementary Material online). The selection of deletion mutants assayed in the original publication was guided by genes having a putative role in the regulation of gene expression, localization to the nucleus, and capacity to modify other proteins, enriching the set for genes that are likely to play a role in the gene regulatory network (Kemmeren et al. 2014).

**Fig. 1.**
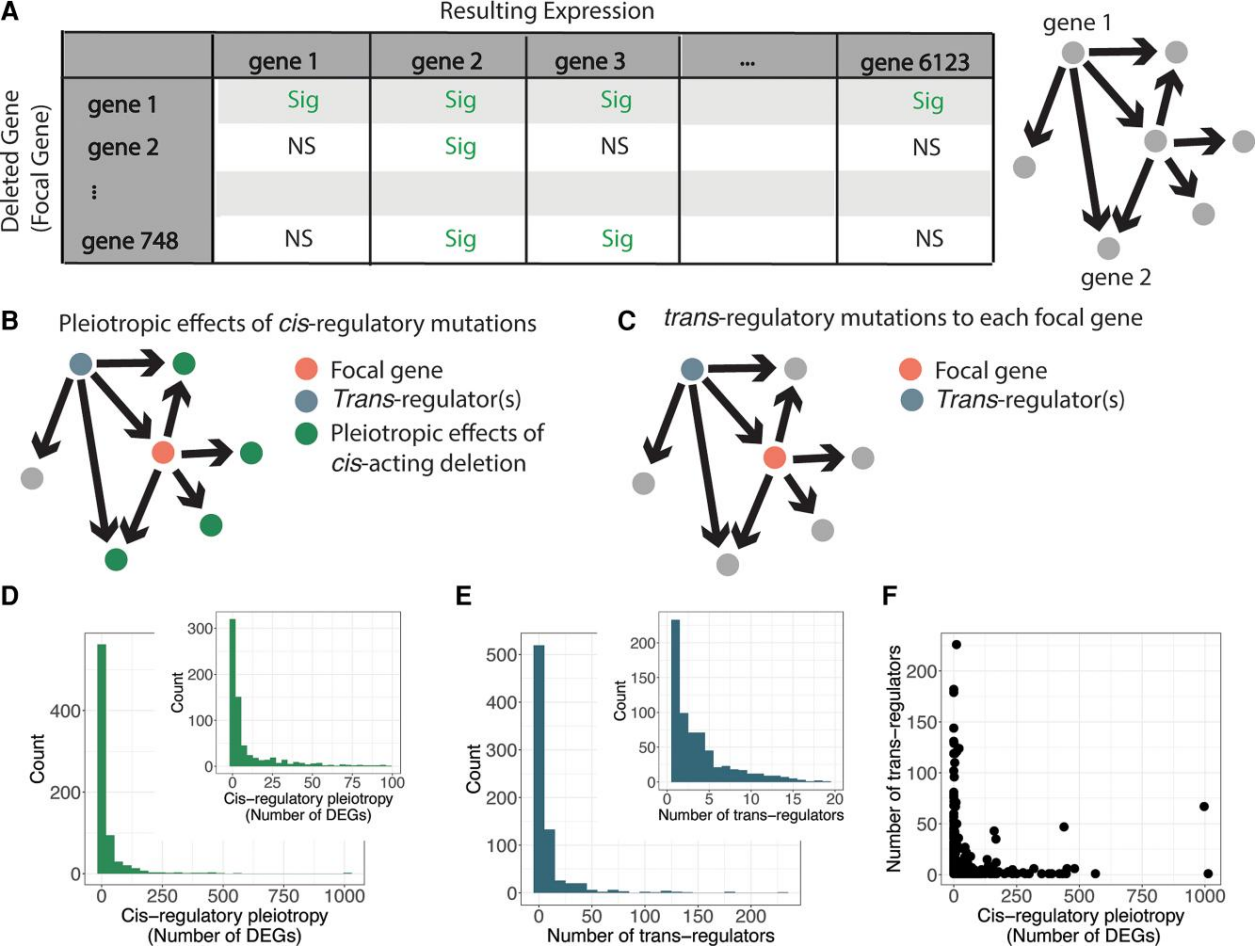
Identification of *cis-* and *trans-*regulatory mutations affecting expression of 748 focal genes in *Saccharomyces cerevisiae*. (*A*) Schematic shows a cartoon example of the perturbation network constructed from gene expression data in *S. cerevisiae* gene deletion mutants. Directed edges are drawn when a deleted gene (gene 1) causes a significant change in expression of another gene (gene 2, among others). In the sample matrix, “Sig” indicates a gene that was significantly differentially expressed in that gene deletion, and “NS” indicates a gene that was not significantly differentially expressed in that gene deletion. (*B*) A copy of the network schematic from figure 1*A* is shown illustrating the pleiotropic effects (green) of a *cis*-regulatory deletion for a gene considered as the focal gene (orange node). (*C*) A second copy of the network schematic from figure 1*A* showing the focal gene in orange and a *trans*-regulatory deletion influencing the expression of the focal gene in blue. (*D*) Histograms show the distribution of pleiotropic effects for *cis*-regulatory deletions, measured as the number of genes significantly differentially expressed upon deletion of the focal gene, for all focal genes. Inset histogram shows close-up of the *x*-axis ranging from 0 to 100 for better resolution of that portion of the data. (*E*) Histograms show the number of deletions that affect expression of each focal gene in *trans* (i.e., the number of *trans*-regulatory deletions for each focal gene). Inset histogram is a close-up of the larger histogram with the *x*-axis ranging from 0 to 20 for better resolution of that portion of the data. (*F*) The number of *trans*-regulatory deletions for each focal gene is plotted on the *y*-axis relative to the number of differentially expressed genes upon deletion of the focal gene on the *x*-axis.

These data can be visualized as a network in which each gene in the data set is represented by a node, and edges are directed from gene 1 to gene 2 when the expression of gene 2 is significantly changed upon deletion of gene 1 (fig. 1*A*). When visualized in this way, the outgoing edges from each deletion strain, or focal gene, represent the effects of a *cis*-acting gene deletion, while the incoming edges identify the deletions with *trans-*regulatory effects on expression of that focal gene (fig. 1*B* and *C*). The deletion of the focal gene itself acts in *cis* to the focal gene because it causes an allele-specific absence of expression for that gene. A complete deletion of the focal gene is expected to cause a more extreme change in expression than other mutations in *cis*-regulatory sequences that modify the focal gene's expression level, but because deletions tend to cause more severe phenotypes than other reductions in expression (Keren et al. 2016), *cis-*acting deletions are expected to be more pleiotropic than other types of *cis*-regulatory mutations. Therefore, using deletions as *cis*-regulatory mutations is a conservative way to test the hypothesis that *cis*-regulatory mutations tend to be less pleiotropic than *trans-*regulatory mutations. Thus, we used the number of genes significantly differentially expressed in the deletion of the focal gene, excluding the focal gene itself, as a simple measure of the pleiotropic effects of a *cis*-regulatory mutation for each focal gene (fig. 1*B*). The distribution of pleiotropic effects of *cis*-regulatory mutations for our 748 focal genes ranged from 0 to 1,013 and was heavy tailed, with many focal genes resulting in minimal gene expression changes across the genome, while relatively few focal genes had widespread effects across the genome (fig. 1*D*).

We then identified sets of deletions with *trans*-regulatory effects on expression of each focal gene as the set of deletion mutants in which the focal gene is significantly differentially expressed. Such genes appear in the network as nodes with an outgoing edge to the focal gene (fig. 1*C*). The number of *trans-*regulators per focal gene ranged from 1 to 226, with a large number of focal genes being influenced by few *trans*-regulators, and a few focal genes being influenced by a large number of *trans*-regulators (fig. 1*E*). Because they are identified exclusively from expression data, these *trans-*regulators can influence expression of the focal gene either directly or indirectly. Therefore, the *trans*-regulators include not only transcription factors directly controlling transcription of the focal gene, but also genes that influence the activity or abundance of transcription factors, including genes affecting cellular systems such as metabolism or progression through the cell cycle, which can indirectly change expression of the focal gene. Effects of *trans*-acting deletions on expression of the focal gene tended to be smaller than the impact of the *cis*-acting deletion of the focal gene itself (supplementary fig. S1, Supplementary Material online). No significant correlation was observed between the number of *trans*-regulatory genes affecting each focal gene and the *cis*-regulatory pleiotropy for the focal gene. In other words, genes with high in-degree (i.e., many *trans*-regulators) were not the same genes as genes with a high out-degree (i.e., influenced the expression of many genes) (fig. 1*F*). Consequently, focal genes whose *cis*-regulatory mutations had many pleiotropic effects were not often the targets of many *trans*-regulators, and focal genes whose *cis*-regulatory mutations had few pleiotropic effects were frequently the targets of many *trans*-regulators.

### *Trans*-regulatory Mutations are More Pleiotropic Than *cis*-regulatory Mutations for Most Focal Genes

**Fig. 2.**
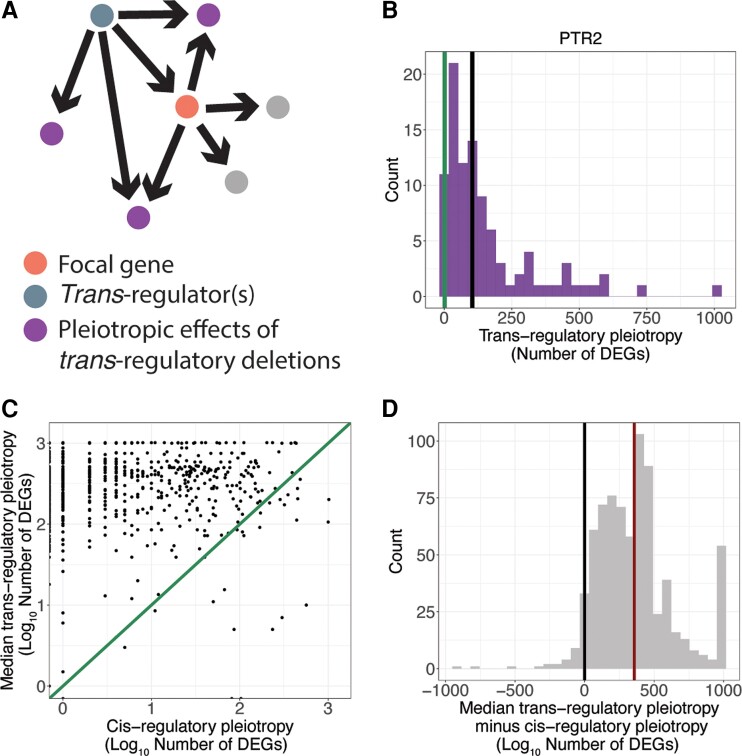
*Cis*-regulatory deletions are less pleiotropic than *trans*-regulatory deletions. (*A*) A copy of the schematic shown in figure 1*A* shows the pleiotropic effects (purple) of a *trans*-regulatory deletion (blue node) to the focal gene (orange node). The pleiotropic effects include all genes differentially expressed in the deletion of the *trans*-regulator, excluding the focal gene and the *trans*-regulator itself. Note that an arrow is shown from the *trans*-regulator to every gene whose expression was altered when the *trans*-regulator was deleted. Consequently, the two gray dots connected to the focal gene shown in pink did not show a change in expression in the *trans*-regulatory deletion of the focal gene shown in blue, and are not considered pleiotropic effects of the *trans*-regulatory deletion. (*B*) The number of differentially expressed genes in the deletion of *PTR2* (pleiotropy of its *cis*-regulatory deletion, green line) is smaller than the median (black line) number of differentially expressed genes for all *trans*-regulatory deletions to *PTR2* (histogram). (*C*) For all focal genes, the log_10_ median pleiotropy of all deletions that act in *trans* to that gene is plotted on the *y*-axis and the log_10_ pleiotropy of the deletion that acts in *cis* to that gene is plotted on the *x*-axis. An *x* = *y* line is shown. (*D*) A histogram of differences between *cis-*regulatory pleiotropy and the median *trans-*regulatory pleiotropy for all focal genes included in the study is shown. The median difference (red line) is significantly greater than zero (black line) (*P*-value = 3 × 10^−165^, one-sided *t*-test).

Having identified sets of *trans*-regulatory mutations affecting expression of each focal gene, we next compared the pleiotropic effects of the *cis*-regulatory deletion of the focal gene to the pleiotropic effects of *trans*-regulatory deletions for that focal gene. We calculated the pleiotropic effects of each *trans*-regulatory mutation as the number of genes significantly differentially expressed upon deletion of the *trans*-regulator, excluding the *trans*-regulator itself and the focal gene (fig. 2*A*). For example, when the membrane peptide transporter *PTR2* was deleted, two other genes were significantly differentially expressed, so the *cis*-regulatory pleiotropy is 2 (fig. 2*B*, green line). There are 96 gene deletions in which *PTR2* is significantly differentially expressed, so there are 96 different *trans*-regulatory mutations for *PTR2*. The total number of genes that are significantly differentially expressed in each of those 96 *trans*-acting mutations, minus the focal gene and *trans*-regulator itself, make up the distribution of the pleiotropic effects of all *trans*-regulatory deletions for the gene *PTR2* (fig. 2*B*, purple histogram).

To compare the pleiotropic effects of *cis*- and *trans*-regulatory deletions for each focal gene, we calculated the median pleiotropy of all *trans-*regulatory deletions for each focal gene (for *PTR2* = 104, fig. 2*B*, black line) and plotted these against the *cis-*regulatory pleiotropy for the same focal gene (fig. 2*C*). Points falling above the *x* = *y* line are focal genes in which the median pleiotropy of the *trans-*regulatory deletions for that focal gene was larger than the pleiotropy of the *cis-*regulatory deletion. We next calculated the difference between the median pleiotropy for the *trans*-regulatory deletions for each focal gene and the pleiotropy of the *cis*-regulatory deletion of the same focal gene. We examined the distribution of differences between *cis-* and *trans-*regulatory deletions for all focal genes (fig. 2*D*) and found that the median of this distribution was significantly greater than zero (Student's *t*-test *P*-value = 3 × 10^−165^), indicating greater pleiotropy of *trans*- than *cis*-regulatory deletions. In fact, only ∼4% of all focal genes (30/748) had a *cis*-regulatory deletion with higher pleiotropy than the median *trans-*regulatory deletion. When considering each *trans-*regulatory deletion individually rather than summarizing the *trans-*regulatory deletions in a median value (supplementary fig. S2, Supplementary Material online), only ∼5% of all *trans-*regulatory and *cis-*regulatory deletion pairs (377/7,162) had more pleiotropic *cis*-regulatory deletions. These results were robust to changes in both the significance and fold-change cutoffs used to identify genes as significantly differentially expressed (supplementary fig. S3, Supplementary Material online).

Measuring pleiotropy as the number of genes differentially expressed does not incorporate information about the magnitude of effect on each gene's expression, so we also calculated the pleiotropy of each gene deletion as the distance from wild-type in multidimensional gene expression space using the Euclidean distance between the deletion and the wild-type. Euclidean distances allow us to incorporate the magnitude of expression change into the measure of pleiotropy, remove the need for significance cutoffs, and create a continuous scale of pleiotropy rather than a discrete scale. The distribution of *cis*-regulatory pleiotropy measured as Euclidean distance was also heavy tailed (supplementary fig. S4*A*, Supplementary Material online). We then compared the pleiotropic effects of *cis*- and *trans*-regulatory deletions for each focal gene using Euclidean distances as the metric of pleiotropy, and found that once again only ∼4% (32/748) of focal genes had greater *cis*-regulatory Euclidean distance than median *trans*-regulatory Euclidean distances (supplementary fig. S4*B*, Supplementary Material online), and only ∼5% of all *trans-*regulatory and *cis-*regulatory deletion pairs had greater *cis*-regulatory Euclidean distance (377/7,162, supplementary fig. S4*C*, Supplementary Material online). Therefore, whether pleiotropy was measured using the number of differentially expressed genes or Euclidean distances, *cis-*regulatory deletions were found to be less pleiotropic than *trans-*regulatory deletions affecting expression of the same focal gene, even though the *cis-*regulatory deletions had greater impacts on expression of the focal gene.

### Nested Effects Do Not Explain Greater *Trans*-regulatory Pleiotropy Than *Cis*-regulatory Pleiotropy

**Fig. 3.**
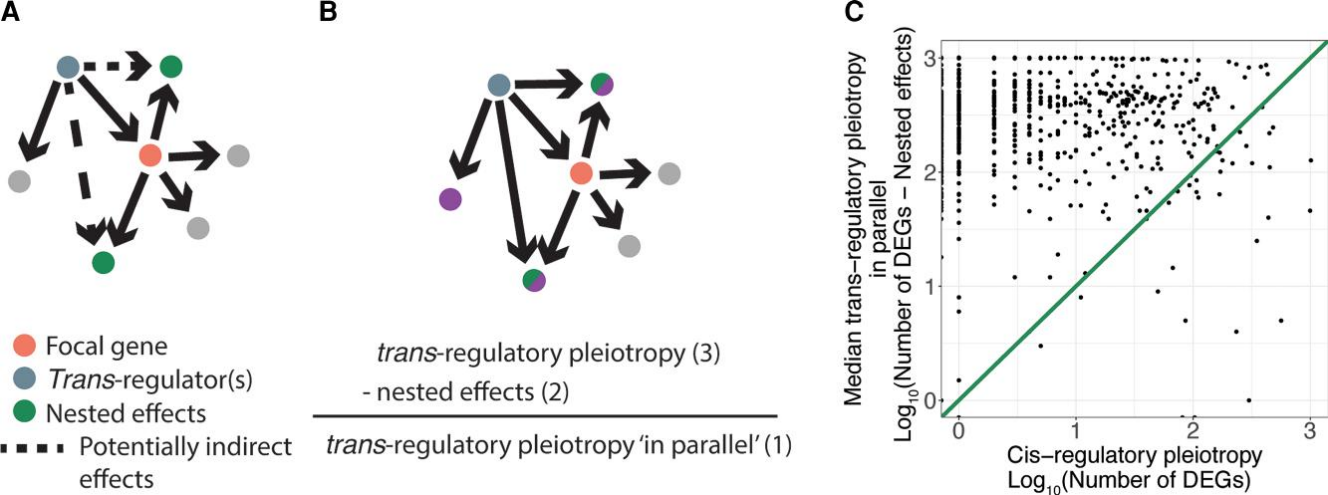
Nested effects do not explain the greater pleiotropy of *trans*-regulatory deletions relative to *cis*-regulatory deletions. (*A*) A copy of the schematic from figure 1*A* shows the effects of the *trans*-regulatory deletion (blue node) that may be due to indirect effects via a change in the expression of the focal gene (orange node). Recall that an arrow is shown from the *trans*-regulator to every gene whose expression was altered when the *trans*-regulator was deleted. Consequently, the two gray dots connected to the focal gene shown in pink in (*A*) did not show a change in expression in the *trans*-regulatory deletion of the focal gene shown in blue and were thus not part of the nested effects observed. (*B*) A copy of the schematic from figure 1*A* shows how nested effects were subtracted from the pleiotropic effects of the *trans*-regulators to estimate pleiotropic effects of *trans*-regulators that occur in parallel to the focal gene. (*C*) The median pleiotropic effects of *trans*-regulators occurring in parallel to the focal gene, calculated as the number of differentially expressed genes in the *trans*-regulatory deletion minus any nested effects, is plotted on the *y*-axis relative to the total pleiotropic effects of the *cis*-regulatory deletion plotted on the *x*-axis. An *x* = *y* line is shown.

We next sought to understand what could explain such a strong pattern of greater pleiotropy for *trans*-regulatory than *cis*-regulatory deletions for the vast majority of focal genes considered. One possible explanation is that the effects of *cis*-regulatory mutations are “nested” within the effects of *trans*-regulatory mutations. In this scenario, *trans*-regulatory mutations affect the expression of the focal gene which in turn affects the expression of downstream genes, making these genes appear as a “nested” portion of the total effects of the *trans*-regulatory mutation (fig. 3*A*). If this “nested effect” is primarily responsible for the greater pleiotropy of *trans*-regulatory mutations relative to *cis*-regulatory mutations affecting expression of the same focal gene, the pleiotropic effects of *trans*-regulatory mutations that occur solely “in parallel” to the effects on the focal gene might not be larger than the pleiotropy of a *cis*-regulatory mutation affecting the focal gene. We therefore removed nested effects from the *trans*-regulatory deletions by excluding genes with significant expression changes in the corresponding *cis-*regulatory deletion. We then compared the pleiotropy of *cis*-regulatory deletions to the pleiotropic effects occurring in parallel only in *trans*-regulatory deletions (fig. 3*B*). We found that only ∼5% (36/748) of all focal genes showed greater *cis*-regulatory pleiotropy than median *trans*-regulatory pleiotropy in parallel (fig. 3*C*), and only ∼6% (413/7162) of all *trans-*regulatory and *cis-*regulatory deletion pairs had greater *cis*-regulatory pleiotropy. We therefore conclude that the inclusion of “nested” effects within the pleiotropy of *trans*-regulatory deletions does not explain the greater pleiotropic effects of *trans*-regulators relative to *cis*-regulatory deletions for the same focal gene. Rather, the pleiotropic effects of *trans*-regulatory deletions that occur solely in parallel to the focal gene alone are greater than the pleiotropic effects of the focal gene deletion itself.

Because gene deletions were used in this study, all *cis*-regulatory deletions decreased (eliminated) the focal gene's expression, but *trans*-regulatory deletions could either increase or decrease expression of the focal gene. The set of expression changes due to an increase in focal gene expression could be very different from those that result from reduction or deletion of focal gene expression. Therefore, we performed an additional analysis that limited our *trans*-regulatory deletions to those that decreased expression of the focal gene, reducing the number of *trans*-regulator:focal-gene pairs to 2,257. We then compared the pleiotropy “in parallel” of the remaining *trans*-regulatory deletions to the *cis*-regulatory deletions for each focal gene. We found that only 9% of these pairs (192/2,257) showed greater *cis*-regulator pleiotropy than *trans*-regulatory pleiotropy “in parallel.” Similar patterns were observed for other measures of pleiotropy such as the total number of differentially expressed genes and Euclidean distances (supplementary fig. S5, Supplementary Material online), showing that the greater pleiotropy observed for *trans*-regulatory deletions is not a consequence of *trans*-regulatory deletions uniquely causing increased expression of the focal gene.

### Network Degree Distribution Can Explain Greater *Trans*-regulatory Pleiotropy

We next wondered whether the degree distribution of the regulatory network as a whole could generate the pattern of greater pleiotropy for *trans*-regulatory deletions relative to *cis*-regulatory deletions affecting expression of the same focal gene. The heavy-tailed distribution of outgoing edges in the network means that there are a relatively small number of highly pleiotropic genes in the network. Many focal genes are likely to be the target of one or more of these highly pleiotropic *trans*-regulators because of the very fact that the *trans-*regulator is highly pleiotropic. In contrast, less pleiotropic *trans*-regulators influence the expression of fewer focal genes, and are therefore unlikely to serve as a *trans*-regulatory mutation for many focal genes. In this way, the out-degree distribution of the regulatory network could inherently generate a pattern in which each focal gene is likely to be influenced by genes that are more pleiotropic than itself. If this is the case, the distribution of outgoing edges in the network should be able to generate the pattern of more pleiotropic *trans*-regulatory than *cis*-regulatory deletions without requiring specific connections between *trans*-regulators and focal genes.

**Fig. 4.**
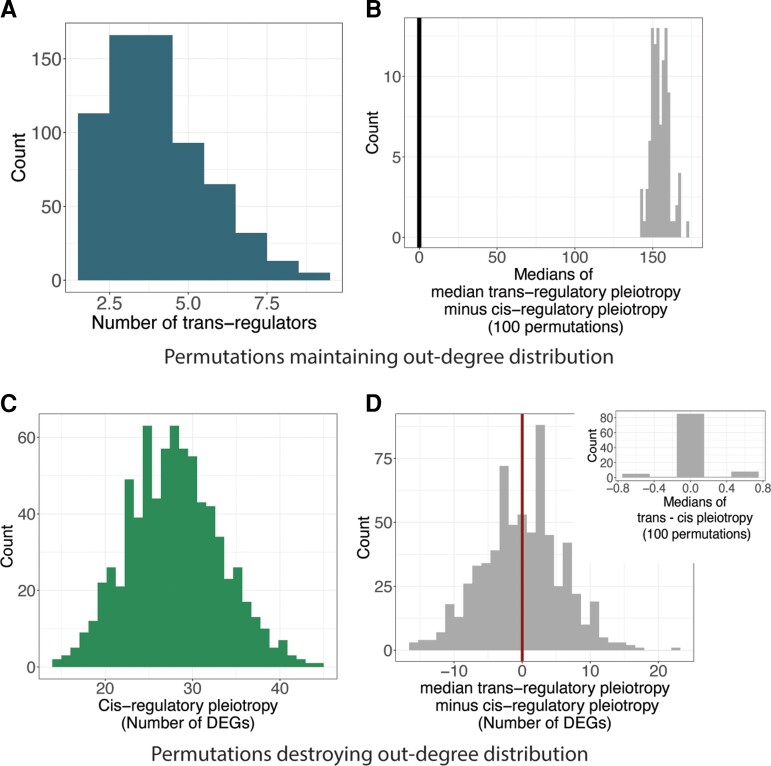
Network topology can explain *trans*-regulatory deletions tending to be more pleiotropic than *cis*-regulatory deletions for most focal genes. (*A*) After permuting all network edges while maintaining the overall out-degree distribution, the number of *trans*-regulators targeting each focal gene is shown in a histogram. In this one permutation, the number of *trans*-regulators per focal gene ranges from 2 to 9. (*B*) After the network edges were permuted (100 permutations) to change connections between individual genes while maintaining the overall degree distribution, all 100 medians of the distributions of differences between the pleiotropy of the *cis-*regulatory deletion and the median pleiotropy of *trans-*regulatory deletions affecting expression of the same focal genes were higher than zero, showing the greater median pleiotropy of *trans*- than *cis*-regulatory deletions in all 100 permuted networks. (*C*) Permuting edges of the perturbation network without maintaining the degree distribution results in distributions of pleiotropy for *cis-*regulatory deletions (green) that are no longer heavy-tailed. Results from one permutation are shown as a histogram. (*D*) The distribution of differences between median *trans*-regulatory pleiotropy and *cis*-regulatory pleiotropy for the same focal gene (histogram) is no longer significantly different from zero (Welch two-sample *t*-test, *P*-value = 0.8221). The large panel shows the result from one permutation. This type of permutation was conducted 100 times. In 98 cases, the median difference between the pleiotropy of the *cis*-regulatory deletion and the median pleiotropy of the *trans*-regulatory deletion was zero, in one case it was −0.5, and in one case 0.5 (inset).

To test whether the network degree distribution rather than the specific connections between nodes are responsible for the difference in pleiotropy between *cis*- and *trans*-regulatory deletions, we permuted the edges of the network in two different ways. In the first, we rearranged all network edges but maintained the overall degree distribution, so that the number of outgoing edges from each node did not change, but the number and identity of *trans*-regulatory mutations for a focal gene did change. For example, for one permutation, the number of *trans*-regulators per focal gene ranged from 2 to 9 (fig. 4*A*). These permutations did not affect the pattern of *trans*-regulatory mutations being more pleiotropic than *cis*-regulatory mutations (fig. 4*B*). In the second type of permutation, we randomly permuted all edges without maintaining the out-degree distribution, changing both the number of outgoing edges from each gene and which genes they were connected to. This changed the overall distribution of outgoing edges so that it was no longer a heavy-tailed distribution (fig. 4*C*). In this case, the difference between the number of differentially expressed genes in *cis*- and *trans*-regulatory deletions was eliminated (fig. 4*D*), indicating that *trans*-regulatory deletions no longer tended to be more pleiotropic than *cis*-regulatory deletions affecting expression of the same focal gene. Consequently, we conclude that the out-degree distribution of the *S. cerevisiae* perturbation network can explain the pattern of less pleiotropic effects of *cis*-regulatory deletions than *trans*-regulatory deletions we observed, and that this trend does not require specific connections between regulators and target genes.

### *Trans*-regulatory Deletions Are More Detrimental to Fitness Than *Cis*-regulatory Deletions Affecting Expression of the Same Focal Gene

**Fig. 5.**
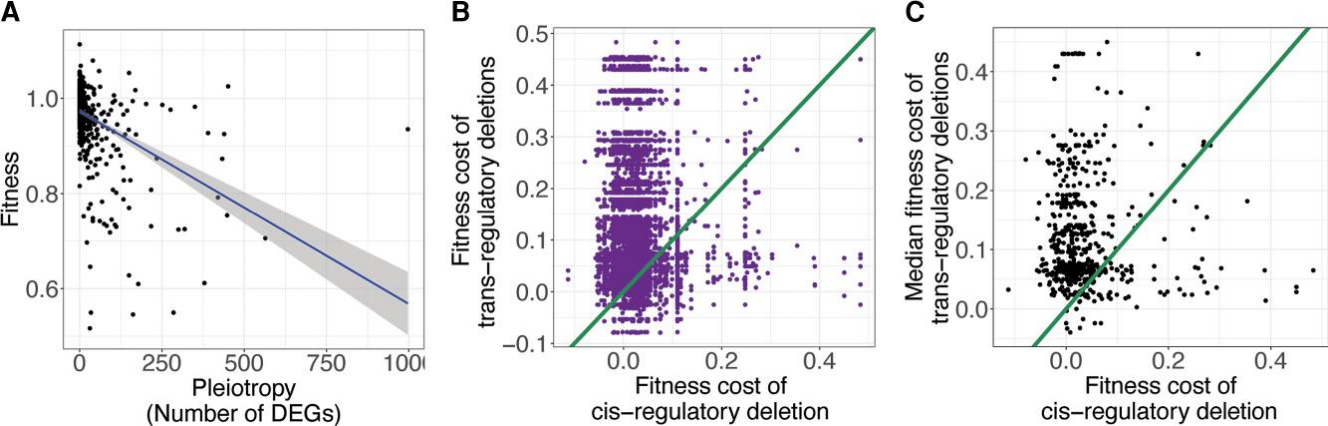
*trans*-regulatory deletions tend to decrease fitness more than *cis*-regulatory deletions that affect expression of the same focal gene. (*A*) The fitness of each gene deletion strain (Maclean et al. 2017) is plotted against the log_10_ number of significantly differentially expressed genes attributable to the deletion of that gene (Kemmeren et al. 2014). The fit line is a least-squares regression surrounded by the 95% confidence interval. (*B*) For every focal gene, one minus the fitness of each *trans*-regulatory deletion (signifying the fitness cost of the mutation) is plotted on the *y*-axis and the fitness cost of the *cis*-regulatory deletion is plotted on the *x*-axis. Each focal gene thus has one *x* value and many *y* values. An *x* = *y* line, corresponding to equal fitness of *cis-* and *trans*-regulatory deletions, is shown. (*C*) For all focal genes, the median fitness cost of all *trans-*regulatory deletions for that gene is plotted on the *y*-axis and the fitness cost of the *cis-*regulatory deletion is plotted on the *x*-axis. An *x* = *y* line is shown.

The differences in the pleiotropic effects of *cis*- and *trans*-regulatory deletions described above may result in a difference in the average fitness effects of these different types of mutations, potentially contributing to a preferential fixation of *cis*-regulatory alleles over time. Consistent with this hypothesis, pleiotropy, defined here as the number of genes differentially expressed as a result of a particular mutation, has been shown to be negatively correlated with fitness in *S. cerevisiae* (Featherstone and Broadie 2002; Vande Zande et al. 2022). The negative correlation suggests that the higher pleiotropy of *trans*-regulatory than *cis*-regulatory deletions may also produce a pattern of lower fitness of *trans*-regulatory deletions when compared with *cis*-regulatory deletions affecting expression of the same focal gene. To test whether this was true for the set of focal genes examined in this study, we used measures of competitive fitness for the same *S. cerevisiae* deletion strains from another study (Maclean et al. 2017). In this study, gene deletion strains were grown together along with a reference strain in rich media and changes in their relative abundance over time were used to calculate their competitive fitness. We found that gene expression pleiotropy as measured from the Kemmeren et al. (2014) data set was significant, although weakly, negatively correlated with fitness measures from Maclean et al. (2017) for all gene deletions present in both data sets (fig. 5*A*, *n* = 1321, *R*^2^= 0.20, *P*-value < 2.2 × 10^−16^). We next examined the fitness cost of *trans*-regulatory deletions relative to the *cis*-regulatory deletions affecting expression of the same focal gene for all focal genes with at least one *trans*-regulator present in both data sets (583/748). We found that only 21% (1,034/4,985) of pairs of *cis*- and *trans*-regulatory deletions affecting expression of the same gene fell below the *x* = *y* line (fig. 5*B*), indicating that the *cis*-regulatory deletion had a greater fitness cost than the *trans*-regulatory deletion. This pattern was stronger when the effects of all *trans*-regulatory deletions affecting expression of a focal gene were represented by the median *trans*-regulatory fitness measure for each focal gene (94/583, ∼16%, fig. 5*C*). These results were robust to different significance thresholds and fold-change cutoffs used to identify *trans*-regulators for each focal gene (supplementary fig. S6, Supplementary Material online). Taken together, these data show that for most focal genes analyzed here, *trans*-regulatory deletions tend to be more deleterious than *cis*-regulatory deletions, consistent with the observation that mutations affecting expression of a focal gene in *cis* seem to be preferentially fixed over evolutionary time.

However, the correlation between fitness and gene expression pleiotropy does not necessarily indicate that greater changes in global gene expression are responsible for reduced fitness. The opposite could also be true: gene deletions with stronger impacts on fitness could reflect changes in growth rate that cause changes in expression of many genes across the genome. Such changes in gene expression that correlate with growth rate across many varied perturbations have been identified by O’Duibhir et al. (2014). These changes were found to be related to a shift in the proportion of cells in the G1 phase of the cell cycle due to slower growth. This transcriptional “slow growth signature” is present in the Kemmeren et al (2014) data set and others. Changes in gene expression attributable to slow growth can be mathematically removed from the data and Euclidean distances re-calculated to estimate the pleiotropic effects of *cis-* and *trans*-regulatory deletions without the consequences of slow growth influencing the pleiotropy estimates. If the highly pleiotropic *trans*-regulators were simply causing large and widespread gene expression changes as a consequence of inducing slow growth, we would expect their Euclidean distances to be dramatically reduced and potentially no longer larger than the *cis*-regulatory deletion affecting expression of the same focal gene that did not induce slow growth. We found that re-calculating pleiotropy as Euclidean distances after subtracting the slow-growth transcriptional signature reduced the pleiotropy of some *trans*-regulators, but did not change the overall pattern we observed. Only 4% (29/748) of focal genes had a more pleiotropic *cis*-regulatory deletion than the median pleiotropy of the corresponding *trans*-regulatory deletions, and 6% (457/7162) of all pairs of *cis*- and *trans*-regulatory deletions affecting expression of the same focal gene had a more pleiotropic *cis*-regulatory deletion (supplementary fig. S7*A* and *B*, Supplementary Material online). Removing the transcriptional slow-growth signature also did not remove the correlation between pleiotropy measured as Euclidean distances and fitness (supplementary fig. S7*C*, Supplementary Material online), indicating that the fitness cost for *trans*-regulatory deletions was higher than for the *cis*-regulatory deletion even when this slow growth signature was removed from the calculation of pleiotropy.

## Discussion

Using gene expression data to compare the pleiotropic effects of gene deletions that affect expression of 748 focal genes in *cis* or *trans*, we found that for the vast majority of these focal genes *trans*-regulatory deletions were more pleiotropic than *cis*-regulatory deletions. Furthermore, we found that fitness was anticorrelated with pleiotropy and therefore *trans*-regulatory deletions affecting a focal gene's expression tended to be more deleterious than *cis*-regulatory deletions affecting expression of the same focal gene. Finally, we found that these relative differences in pleiotropy can be explained by the degree distribution of the inferred regulatory network. Together, these findings provide strong support for the hypothesis that pleiotropy plays a key role in the preferential fixation of *cis*-regulatory changes over evolutionary time. They also present other interesting implications for how we might expect gene expression to evolve.

Our finding that the relative difference in pleiotropy between *cis*- and *trans*-regulatory mutations affecting expression of the same focal gene emerges from the overall degree distribution of the regulatory network is akin to the “friendship paradox” described in other contexts as an emergent feature of scale-free networks, which are networks for which the degree distribution follows a power law (Feld 1991; Barabási and Oltvai 2004; Kooti et al. 2014). The friendship paradox describes the observation that nodes are likely to be connected to nodes that are more highly connected than themselves and can be stronger or weaker depending on other network parameters such as assortativity (Cantwell et al. 2021). The heavy-tailed out-degree distribution of the regulatory network of *S. cerevisiae* approximately follows a power law (Featherstone and Broadie 2002; Kemmeren et al. 2014) (supplementary fig. S8, Supplementary Material online) and we find exhibits a strong “friendship paradox.” Consequently, assuming the degree distributions for the genes we analyzed are representative of all genes in the genome, we expect these patterns to hold for most genes. It is possible, however, that the omission of certain types of genes from the data set (e.g., genes that cause lethality when deleted) introduces a bias in the degree distribution. Nonetheless, many biological networks appear to have heavy-tailed degree distributions (Barabási and Oltvai 2004), although not all are strictly scale-free (Broido and Clauset 2019), suggesting that the trends we describe here could be applicable to species beyond *S. cerevisiae*.

Extending this work to think about regulatory variation in other contexts requires keeping a few caveats in mind. First, most variation segregating within a population or fixed between species involves point mutations in the gene body or promoter rather than full gene deletions. Compared with gene deletions, such point mutations are expected to generally have smaller and more varied effects on gene activity (e.g., point mutations can increase a gene's activity whereas a gene deletion cannot). Second, the structure of the regulatory network was inferred here using expression data from a single strain in a single environmental condition, but this structure is not expected to be static among species, within a species, or even within an individual among environments. How regulatory networks differ among environmental conditions (Luscombe et al. 2004) and how they change over time (Siddiq and Wittkopp 2022) remain important open questions that could influence the connections within a regulatory network. That said, we found that permutations of network structure maintaining the degree distribution despite shuffling specific connections (fig. 4*A* and *B*) retain the pattern of *trans*-regulatory mutations being more pleiotropic than *cis*-regulatory mutations affecting expression of the same gene. In other words, even if the identities of highly pleiotropic genes shift over time or among environments, *cis*-regulatory mutations could continue to generally be less pleiotropic than *trans*-regulatory mutations affecting expression of the same gene as long as the general degree distribution is maintained.

Systematic differences in pleiotropy and fitness between *cis*- and *trans*-regulatory mutations affecting expression of the same gene are important because they can influence the molecular mechanisms by which gene expression evolves: a tendency for lower pleiotropy and fitness cost of *cis*-regulatory mutations suggests they might contribute more than *trans*-regulatory mutations to divergence in the focal gene's expression. The greater frequency with which *trans*-regulatory mutations arise, however, might counter-balance this fitness advantage of *cis*-regulatory mutations (Metzger et al. 2016). Each gene is regulated by multiple potential *trans*-regulators, but the set of potential *trans*-regulators “overlaps” for many focal genes such that some *trans*-regulators, specifically those that are the most pleiotropic, are likely to harbor mutations that act in *trans* on expression of many focal genes. However, mutations affecting these highly pleiotropic *trans*-regulators are likely to be detrimental to fitness when mutated, constraining their evolution. Studies comparing the evolution of gene expression levels to properties of network structure have failed to identify consistent patterns (Kopp and McIntyre 2012; Yang and Wittkopp 2017), but it is unclear whether this is because they do not exist or because of issues with the incomplete regulatory networks used for these analyses. Further studies that better define regulatory networks, incorporate multiple properties of regulatory mutations, and consider evolution of all genes in the genome simultaneously are needed to understand how regulatory networks evolve and influence the expression divergence of the genes that compose them.

## Materials and Methods

### Expression Data and Inference of the Perturbation Network

The file containing microarray expression data used in this study to build a perturbation network for gene deletions in *S. cerevisiae* from Kemmeren et al. (2014) named “deleteome_all_mutants_ex_wt_var_controls.txt” was downloaded from http://deleteome.holstegelab.nl/ on June 19, 2020. This file included all *M* values and *P*-values for expression changes of each gene on the microarray (*n* = 6,123) for each gene deletion strain relative to the wild-type control, where the *M* value is the log_2_ fold-change in expression and the *P*-value is obtained after Benjamini–Hochberg FDR correction for a statistically significant change in expression relative to a wild-type strain as calculated using the *limma* R package. *Limma* uses linear expression models and an empirical Bayes model to moderate standard errors and calculate a moderated *t*-statistic and log-odds of differential expression (Smyth et al. 2005). The file also included experiments for strains grown in different media types, which were removed from the data set for the analyses conducted in this paper. The authors of this study were aware of the aneuploid strains that have been identified in yeast gene deletion mutants and analyzed all expression profiles for evidence of aneuploidy. Any strains showing evidence of aneuploidy were remade and re-assayed or excluded from the data set by the original authors. The file was read into R (version 3.5.2) where all statistical analysis was performed. A gene was considered significantly differentially expressed in a deletion mutant if it showed a 1.7 or greater log fold-change in expression and a *P*-value of ≤0.05 after Benjamini–Hochberg FDR adjustment, resulting in a directed edge drawn from the deleted gene to the differentially expressed gene. In this way, we generated a binary, asymmetrical adjacency matrix in which rows represent each deleted gene and columns represent the expression levels of all genes in the microarray. We then excluded genes for which their deletion did not cause a statistically significant decrease in their expression or whose expression was not assayed by the microarray. This matrix was the basis for all analyses on gene expression described in this paper (except supplementary figures assessing the robustness of results to different fold-change or significance cutoffs), and is available as supplementary Table S2, Supplementary Material online. From this matrix, focal genes were identified as those deleted genes whose expression was influenced by at least one other deletion mutant in the matrix. The final list of focal genes, the functional categories they were placed in by Kemmeren et al. (2014), and the number of differentially expressed genes in each deletion mutant are found in supplementary Table S1, Supplementary Material online. To test the robustness of patterns reported in the main text, we repeated analyses with different cutoffs used for both the fold change and *P*-value, with results shown in supplementary figure S3, Supplementary Material online.

### Estimating Pleiotropy as Euclidean Distance From Wild-Type in Multidimensional Gene Expression Space

To estimate pleiotropy as a Euclidean distance from wild-type in multidimensional gene expression space, we used fold changes calculated from the vector of *M* values (log_2_ fold changes) for each gene deletion mutant included in the study without using any fold change or significance cutoffs. Specifically, Euclidean distances were calculated as the square root of the sum of the squared fold changes of all genes measured in the study. This calculates the distance between the deletion mutant and the wild-type strain which is placed at the origin in multidimensional space when each gene's expression constitutes a dimension.

### Assessing the Impact of Network Topology

To determine whether the perturbation network was consistent with a scale-free topology, we tested whether it was well represented by the formula, *p*(*K*)∼ = *K*^−gamma^, where *K* is the node out-degree, or number of edges proceeding from the node. *p*(*K*) was calculated from the empirical distribution of *K* values. The relationship between *p*(*K*) and *K* was then assessed using a least-squares regression of the log(*p*(*K*)) on the log(*K*). The linear regression was highly significant (*R*^2^ = 0.73, *P*-value ≤ 2 × 10^−16^), with a coefficient (which translates to the -gamma value) of −0.75. This value is similar to the gamma of 0.7 calculated for a perturbation network of a smaller yeast gene expression data set (Featherstone and Broadie 2002).

Two types of permutations to the network topology were conducted. In both, the number of nodes and edges was not changed, only the structure of the network itself. In the first type of permutation, conducted 100 times, we randomly reassigned the target of each directed edge without re-assigning the node the edge came from, changing the structure of the network without affecting the out-degree distribution. We did this by randomly shuffling the values of each row of the adjacency matrix described above. For the second type of permutation, also conducted 100 times, we randomly shuffled all edges of the adjacency matrix by first randomly shuffling the values in each row, and then randomly shuffling the values in each column. This resulted in a random network structure as demonstrated by the roughly normal distribution of out-degrees for each node (fig. 4*C*).

### Measures of Fitness for Gene Deletions

Fitness measurements for gene deletion strains are from Maclean et al. (2017) as reported in supplementary Data 6, Supplementary Material online of that publication. The reported fitness measures of gene deletions relative to the reference strain in YPD media were used directly, without any sort of significance cutoff to identify deletions that resulted in a statistically significant decrease in fitness.

### Assessing Pleiotropy of *Cis*- and *Trans*-regulatory Deletions After Removal of a “Slow-Growth Signature”

Gene expression estimates for all genes measured in Kemmeren et al (2014) with the slow-growth signature removed by O’Duibhir et al (2014) named “deleteome_all_mutants_svd_transformed.txt” was downloaded from http://deleteome.holstegelab.nl/ on September 7, 2022. Euclidean distances were calculated as described above from this set of transformed values for each deletion strain included in the analysis.

### Statistical Analyses

All statistical analyses and plots were produced using R (version 3.5.2) with code available on Github at https://github.com/pvz22/Network_pleiotropy.

## Supplementary Material

msac266_Supplementary_Data

## Data Availability

All data analyzed in this work are publicly available from the original sources, as described in Materials and Methods. Code used to analyze the data is available on Github at https://github.com/pvz22/Network_pleiotropy.
