# Supplementary material for: Network Topology Can Explain Differences in Pleiotropy Between Cis- and Trans-regulatory Mutations
Source: Mol Biol Evol. 2022 Dec 12;39(12):msac266. doi: 10.1093/molbev/msac266 (PMC9791367; doi:10.1093/molbev/msac266)
Supplement: msac266_Supplementary_Data [file msac266_supplementary_data.zip › Supplementary Figures.pdf]

Supplementary Figures for "Network topology can explain differences in pleiotropy between *cis* and *trans*-regulatory mutations"

Supplementary Figure 1

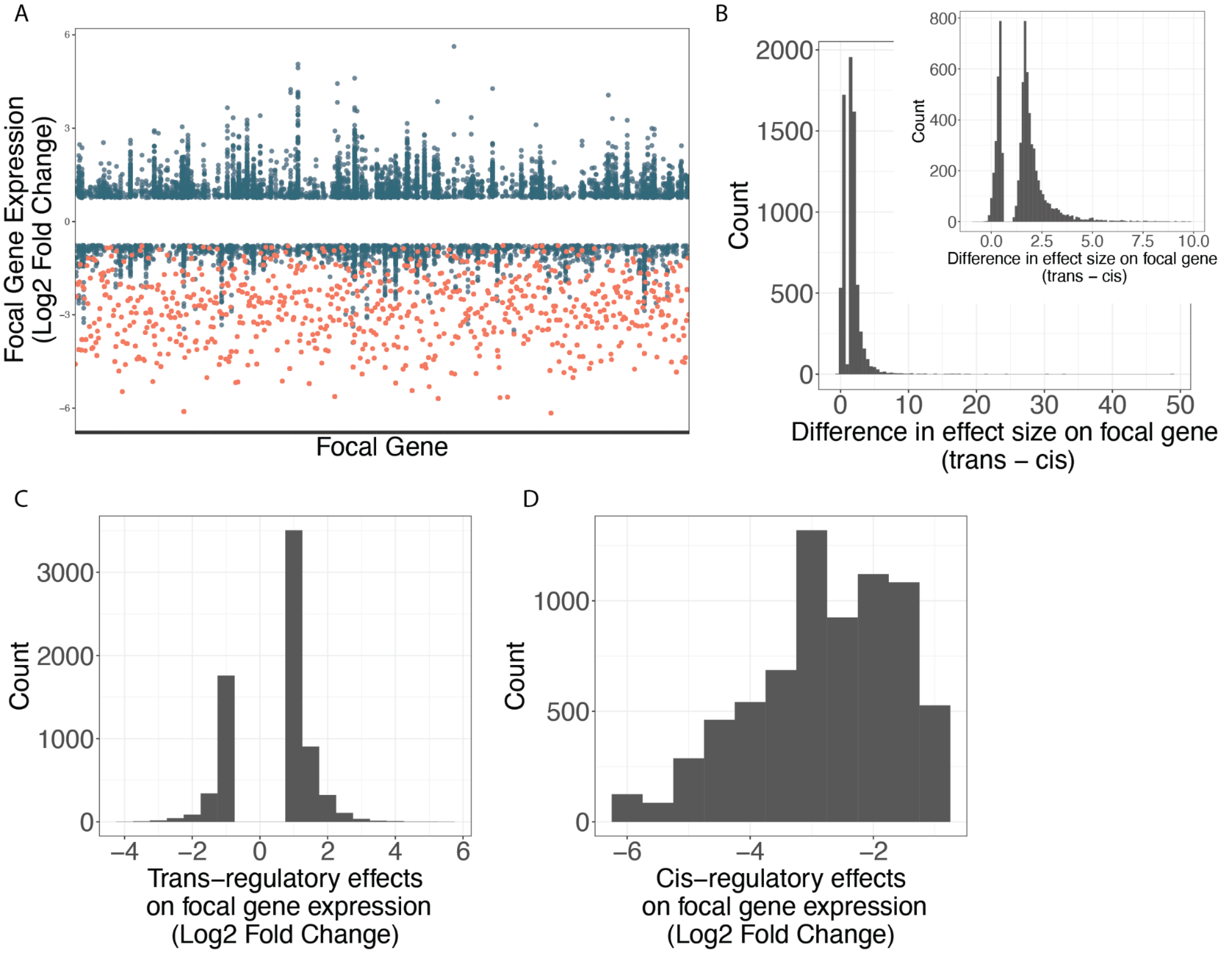

FIG. S1 *cis*-regulatory deletions have larger effects on expression of the focal gene than *trans*-regulatory deletions. *(a)* For each deletion mutant (focal gene, x-axis), the value of the focal gene's expression level relative to the wild-type control is plotted for the deletion mutant itself (*cis*-regulatory deletions, orange points) and all other deletion mutants in which the focal gene was significantly differentially expressed (*trans*-regulatory deletions, blue points). *(b)* A histogram of the difference between the effect size of a *cis*-regulatory deletion and the *trans*-regulatory deletions on expression of the same focal gene. The fold change cut-off used in conjunction with a p-value cutoff to determine *trans*-regulatory deletions with a significant effect on each focal gene's expression generates two peaks in the histogram - one for *trans*-regulatory deletions that increase focal gene expression and one for *trans*-regulatory deletions that decrease

focal gene expression. In ~99% of cases, the *cis*-regulatory deletion is more extremely downregulated than a *trans*-regulatory deletion. For the remaining ~1% of cases, the difference is very close to zero, suggesting that both deletion of the gene and the *trans*-regulatory deletion eliminated expression of the focal gene. Inset shows the same histogram limited on the x-axis from -1 to 10 for better visualization of that range of the data. *(c)* A histogram shows the distribution of all *trans*-regulatory deletions effects on focal gene expression (all blue points in panel A). *(d)* A histogram shows the distribution of all *cis*-regulatory deletions effects on focal gene expression (all orange points in panel B).

Supplementary Figure 2

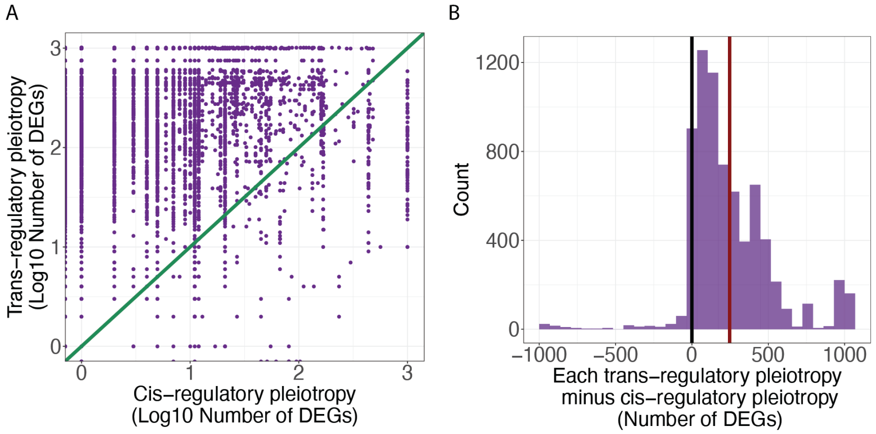

FIG. S2: *trans*-regulatory deletions tend to be more pleiotropic than *cis*-regulatory deletions affecting expression of the same focal gene in pairwise comparisons. *(a)* For every focal gene, the pleiotropy of each *trans*-regulatory deletion is plotted on the y-axis and the pleiotropy of the *cis*-regulatory deletion is plotted on the x-axis. Therefore, for each focal gene there is one x value and many y values. An x=y line is plotted in green. *(b)* A histogram of the differences between the pleiotropy of *cis*- and *trans*-regulatory deletions for all pairs of one *trans*-regulator and the corresponding focal gene (all purple points in panel A). The median (red line) is significantly higher than zero (one-sided t-test, p-value = 0).

Supplementary Figure 3

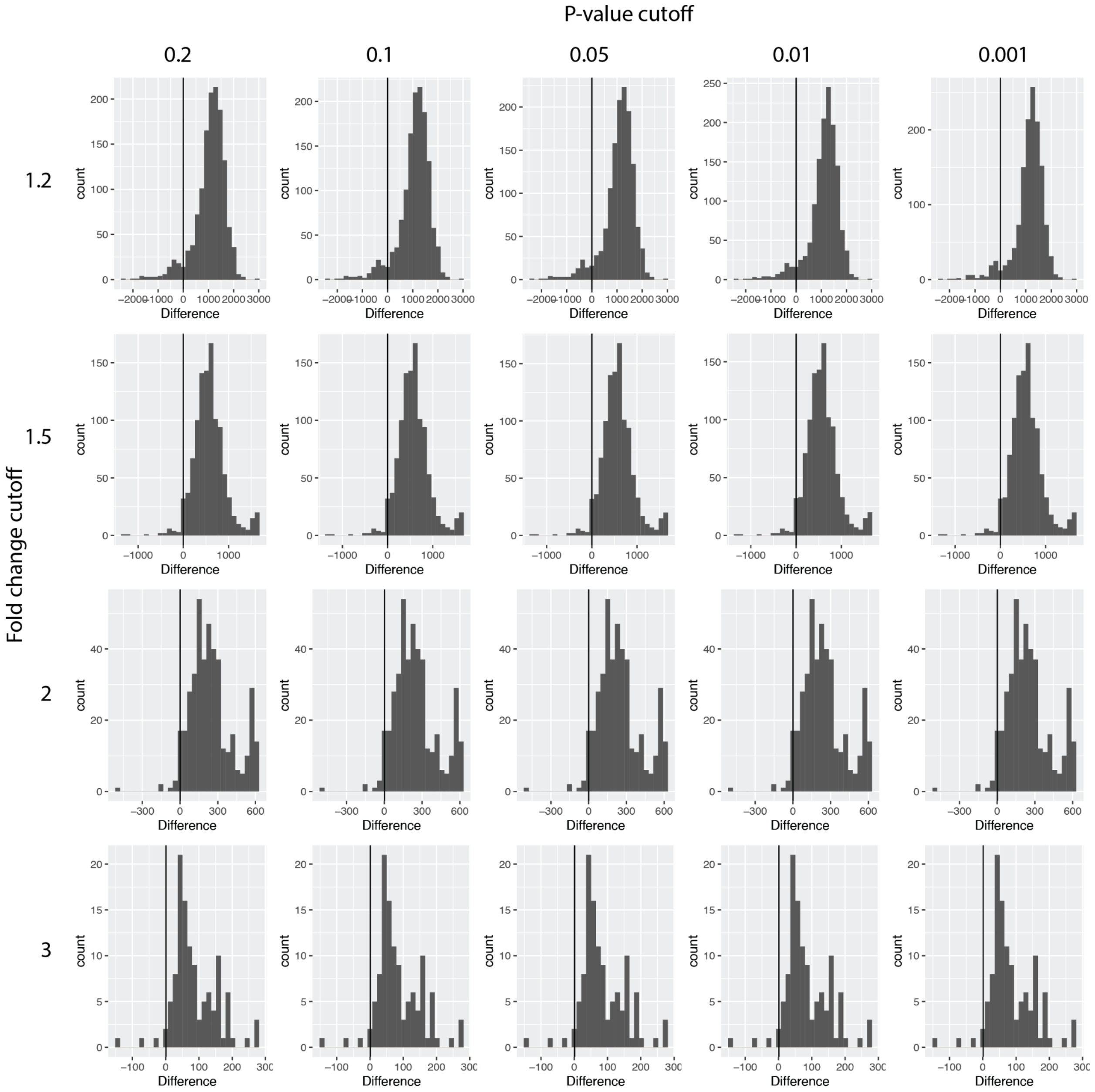

FIG. S3: Difference between median pleiotropy of *trans*-regulatory deletions and *cis*-regulatory deletion for all focal genes is robust to changes in thresholds use to identify significantly differentially expressed genes. Each panel shows the distribution of differences in pleiotropy between the *cis*-regulatory deletion and the median pleiotropy of the *trans*-regulatory deletions affecting expression of the same focal gene. The fold-change cutoffs (rows) and *P*-value cutoffs (columns) used to classify a gene as significantly differentially expressed in each case are shown. The similarity of distributions across columns demonstrates that fold-change cutoffs had a larger impact on the number of edges in the network than *P*-value cutoffs. Despite these effects, the

median of the difference in pleiotropy between the *cis*- and *trans*-regulatory deletions were higher than zero for all cutoff combinations, showing that the *trans*-regulatory deletions tended to be more pleiotropic than the *cis*-regulatory deletion affecting expression of the same focal gene regardless of the criteria used to identify significant changes in gene expression.

Supplementary Figure 4

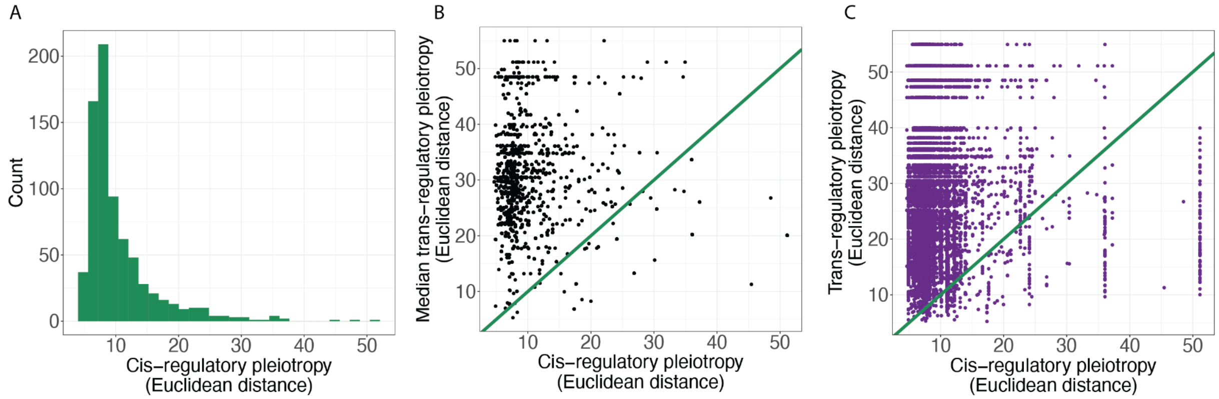

FIG. S4 Pleiotropic effects measured as Euclidean distances between deletion mutants and the wild type strain in multidimensional gene expression space. *(a)* The distribution of Euclidean distances calculated for each deletion of the focal gene (i.e., *cis*-regulatory deletion), is shown as a histogram. *(b)* The median Euclidean distance from wild-type of all *trans*-regulatory deletions to a focal gene is plotted on the y-axis relative to the Euclidean distance of the *cis*-regulatory deletion of the focal gene on the x-axis. An x=y line is shown in green. *(c)* Euclidean distances for each *trans*-regulatory deletion is plotted on the y-axis relative to the Euclidean distance of the *cis*-regulatory deletion of the focal gene on the x-axis. Because each focal gene has one *cis*-regulatory deletion and multiple *trans*-regulators, there are many y values for each x value.

Supplementary Figure 5

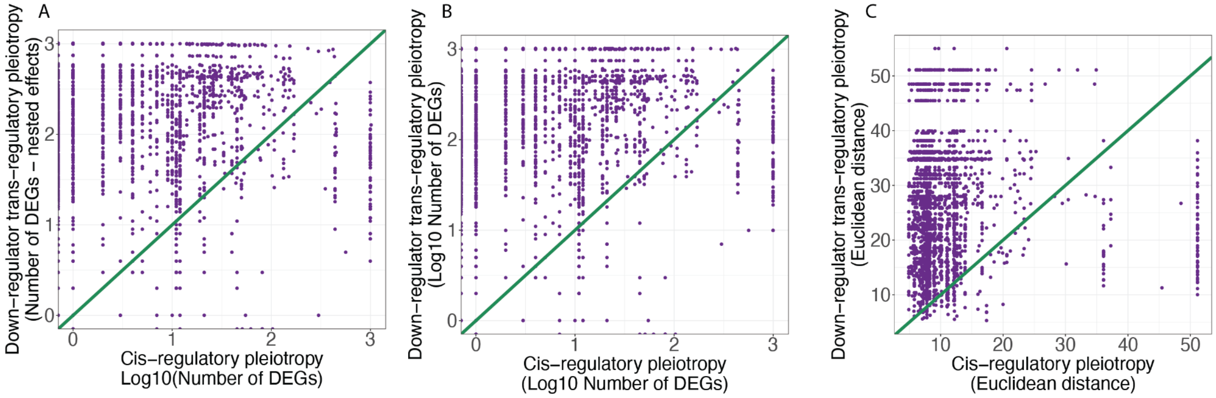

FIG. S5: Limiting *trans*-regulators to those that only decrease focal gene expression results in similar network patterns. *(a)* The ($\log_{10}$) number of differentially expressed genes minus any potential nested effects in each *trans*-regulatory deletion that decreases expression of the focal gene is plotted on the y-axis, and the ($\log_{10}$) number of differentially expressed genes in the *cis*-regulatory deletion of the focal gene on x-axis. *(b)* The ($\log_{10}$) number of differentially expressed genes in each *trans*-regulatory deletion that decreases expression of the focal gene is plotted on the y-axis and the ($\log_{10}$) number of differentially expressed genes in the *cis*-regulatory deletion of the focal gene is plotted on the x-axis. *(c)* The Euclidean distance from wild type for each *trans*-regulatory deletion that decreases the expression of the focal gene is plotted on the y-axis, while the Euclidean distance from wild type for the *cis*-regulatory deletion is plotted on the x-axis.

Supplementary Figure 6

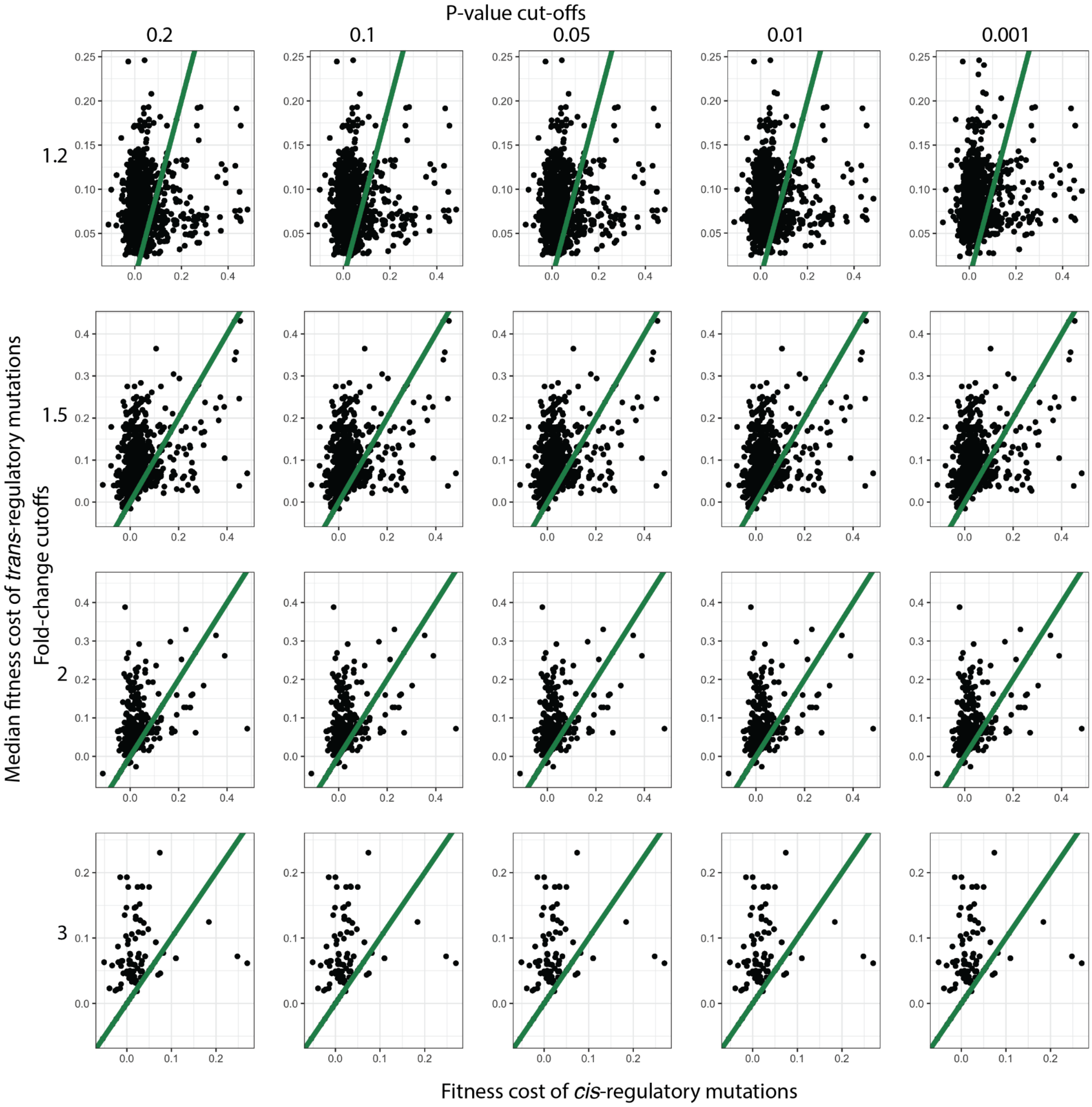

FIG S6: Difference between median fitness cost of *trans*-regulatory deletions and *cis*-regulatory deletion for all focal genes is robust to changes in thresholds use to identify *trans*-regulatory deletions for each focal gene. Each panel shows a scatterplot of median fitness costs (1-fitness) for *trans*-regulatory deletions on the y-axis plotted according to the fitness cost of the *cis*-regulatory deletion for the same focal gene on the x-axis. The fold-change cutoffs (rows) and *P*-value cutoffs (columns) used to classify deletion mutants as a *trans*-regulatory deletion to each

focal gene in each case correspond to the same thresholds shown in Supplementary Fig. 3. Each panel also shows an x=y line in green.

Supplementary Figure 7

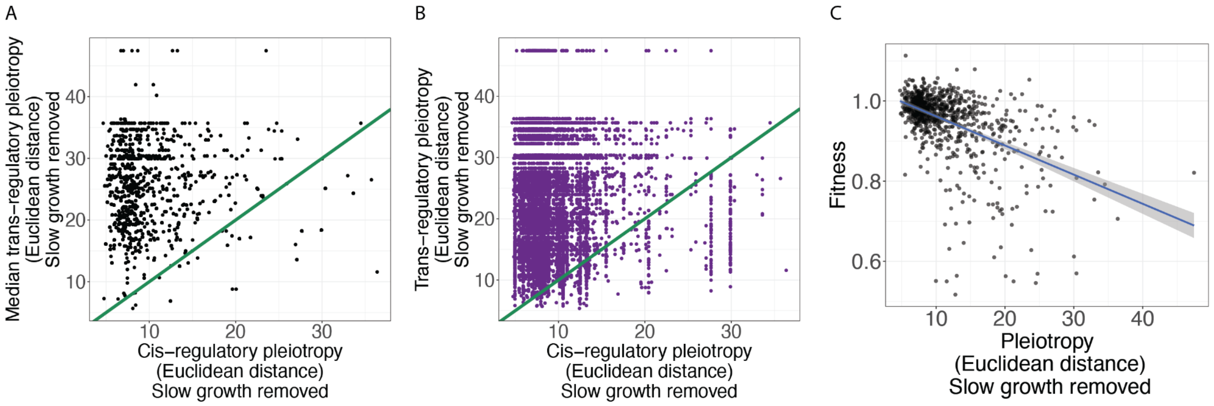

FIG. S7: *trans*-regulatory deletions are more pleiotropic than *cis*-regulatory deletions affecting expression of the same focal gene after subtraction of a transcriptional 'slow growth signature'. *(a)* Median *trans*-regulatory pleiotropy estimated as Euclidean distance from wild type after subtraction of the slow growth signature described in O'Duibhir et al (2014) is plotted on the y-axis against the pleiotropy of the *cis*-regulatory deletion also estimated as Euclidean distance after subtraction of the slow growth signature. *(b)* Same as in panel A, but for all *trans*-regulator:focal-gene pairs rather than median *trans*-regulatory deletion values on the y-axis. *(c)* Fitness is plotted on the y-axis against pleiotropy when measured as Euclidean distance from wild type after subtraction of the slow growth signature on the x-axis. Blue line is a least-squares regression line, with shaded areas showing 95% confidence intervals.

Supplementary Figure 8

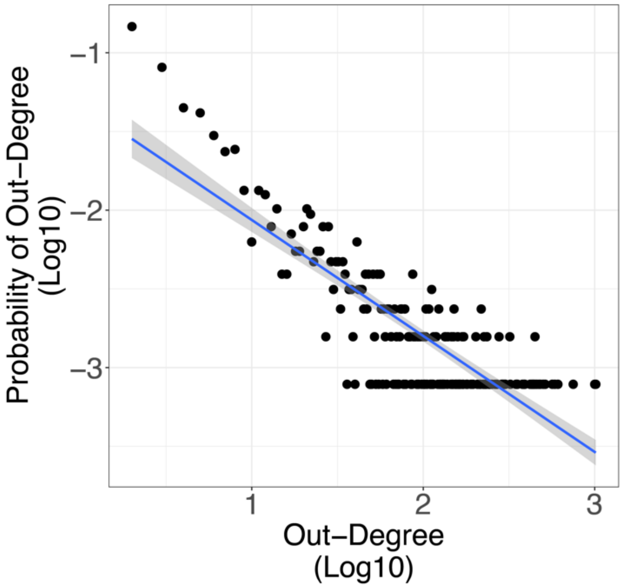

FIG. S8: The out-degree distribution, or number of outgoing edges from each node (gene), follows a power-law distribution, as indicated by a significant linear relationship (black line surrounded by 95% confidence intervals) between the log of the out-degree and the log of the probability of that out-degree (see Methods, $R^2 = 0.73$, p-value = $< 2x10^{-16}$).
